# Supplementary material for: Patterns of Intron Gain and Loss in Fungi
Source: PLoS Biol. 2004 Nov 30;2(12):e422. doi: 10.1371/journal.pbio.0020422 (PMC532390; doi:10.1371/journal.pbio.0020422)
Supplement: Table S1 — Also available at http://genes.mit.edu/NielsenEtAl/. (4.3 MB ZIP). [file pbio.0020422.st001.zip › NielsenEtAl/html/950.html]

AN7141.1.NCU08669.1.MG05814.1.FG09762.1


```
 CLUSTAL W (1.82) Multiple Sequence Alignments - Introns Inserted


Sequence 1: MG05814.1	470 aa
Sequence 2: FG09762.1	467 aa
Sequence 3: NCU08669.1	467 aa
Sequence 4: AN7141.1	470 aa
Alignment Length: 474 aa
Number Identitical Residues: 209 aa
Alignment Score (without introns) 11714


MG05814.1 	MGSVETLTTISPTTNEGILQRTGVSLSELEQLPKTATEAFKSWSKTTLADRQIIIRKALE
NCU08669.1	-MSVEVITTISPTTGEPIITRNGISEEELVQLPETAFKAFQGWRTTKLEDRQIIVKKALK
FG09762.1 	-MSVETITTISPNTNEAILTRNGASAADLESLPKVAAEAFQNYRKTTLKERQAIVRKFLD
AN7141.1  	--MSNQVRTLSPSTGKVLFEHPGVTVEQVRQIAQASEDAFRTYRELSLDQRKAIVVKALE
          	    : : *:**.*.: :: : * :  :: .:.:.: .**: :   .* :*: *: * *.

MG05814.1 	GLSAKKDELANELTVQMGRPIAYTGGEVATAIKRAEYLLKISDEVLKDTDGEAEKGFKRL
NCU08669.1	LLAEKQDELAEELTVQMGRPIAYTAKEVATAIKRSEYLLKISNDALKDTDGEEEKGFKRF
FG09762.1 	GLLAKKDELGEELTVQMGRPISYTPGEVATAVKRGEYLLKISDEALKDTDGEAEKGFKRF
AN7141.1  	IIDANKETLAHELTTQMGRPISYTAGEVDTMRKRANYLIDQAEDALKTIPGQEENGFKRF
          	 :  ::: *..***.******:**  ** *  **.:**:. :::.**   *: *:****:

MG05814.1 	IRKVPVGPVLVIFAWN~----YPYLILVNSLIPALLAGNTVILKPSPQTPTIVEQVAGVF
NCU08669.1	IRKVPVGPVLIIFAWN0----YPYLILVNALIPALLSGNSVILKPSPQTPTIVEQVAKVF
FG09762.1 	IRKVPVGPVLIIFAWN0----YPYLILVNSLIPALLAGCSVILKPSPQTPTIVERVTDLL
AN7141.1  	VKKAPVGPVLLATAWN0LTGQYPYLITINALVPALLAGNTVILRPSPQTPLVGDRLSEYF
          	::*.******:  ***  :..***** :*:*:****:* :***:****** : ::::  :

MG05814.1 	ADAGLPQGVIQYFHCGSPTLMETIVRDPLVKHVCFTGSVAGGLAVQKAASDRIVNVGLEL
NCU08669.1	SEAGLPDGVIQYFHSGSPTVIETIVRNPKIANICFTGSVAGGLAVQKAASDRIVNVGLEL
FG09762.1 	KEAGLPEGVCQYFHCGSPTVMETIVRDPKIELVCFTGSVAGGLAVQQAASDRIVNVGLEL
AN7141.1  	EKAGLPKNVLQVVHLGSWDVLDEVVKIPQIKLVSFVGSTQGGLRLRQATAGRILPLNLEL
          	 .****..* * .* **  ::: :*: * :  :.*.**. *** :::*::.**: :.***

MG05814.1 	GGKDPAYVRSDVDVDWVAGEIVDGAVFNSGQSCCSIERVYVHDDIHDKFVAAVQKVLEG~
NCU08669.1	GGKDPAYVRGDVDIAWAAEEIVDGAVFNSGQSCCSIERVYVDEKIHDEFIAAVQNVLKG~
FG09762.1 	GGKDPAYVRSDVDIDWAAAEIVDGAIFNSGQSCCSLERVYVVEKIHDQFVEAVQKVLKS~
AN7141.1  	GGNDPAYVRADADLAYTAAQVVDGAVFNSGQSCCSIERIYVHADVHDAFVAEVRKELAT2
          	**:******.*.*: :.* ::****:*********:**:**  .:** *:  *:: *   

MG05814.1 	YVLGDPLDKGTHVGPVVSKRSKEAIEAHVKEAIEKGAKDATPKNESFDTVPSSSKGNFVK
NCU08669.1	YKLGDPLDKGTHLGPVISARSKETIQAHIKDALDKGAKNATPENETFSILPD--KGNFVA
FG09762.1 	YKLGDPFNKETQIGPVVSKRSKQAAEEHIKDAVDNGAKDATPENETFSNPPP--KGNFVK
AN7141.1  	YKLGDPLDKATTTGPVISHQAVKNIQAHIDDALSRGAVDSTPENPTFAKIPS--EGSFIA
          	* ****::* *  ***:* :: :  : *:.:*:..** ::**:* :*   *   :*.*: 

MG05814.1 	PTLLVNVNHSMQVMKDETFGPVI~PVMRVKSDEEAVQLMNDSEFGLTASIWTKDTDTAAK
NCU08669.1	PTLLTGVDHTMTVMKDETFGPVI~PVMKVKSDAEAIQLMNDSEFGLTASIWTKDTAKGYE
FG09762.1 	PTLLTGVNHSMRVMTEESFAPII1PVMKVKDDSEAIKYMNDSEFGLTASIWTKDTDKGYE
AN7141.1  	PRVLTNVSHDMRVMREETFGPVV~PIMKVQSDDEAVALMNDSDYGLTASVWTKDIKAGED
          	* :*..*.* * ** :*:*.*:: *:*:*:.* **:  ****::*****:****   . .

MG05814.1 	LIDDVEAGTVFVNRCDYPSP0DLAWTGWKNSGKGQTLSRFGFDQFVKLKSYHMKDYPK
NCU08669.1	LAEEVEAGTVFVNRCDFPSP0DLAWTGWKNSGKGQTLSKYGFDQFVKLKSYHLKDYPK
FG09762.1 	LAEEVEAGTVFVNRCDYPAP0DLAWTGWKNSGKGQTLSKFGFDQFVKLKSYHLKDYPN
AN7141.1  	LIERIEAGTVFINRCDYPSP0DLAWIGWKSSGLGCSLGPQAFDAFYKLKSFHIRTTHG
          	* : :******:****:*:* **** ***.** * :*.  .** * ****:*::
```
